# Designing and Validating a Novel Method for Assessing Delay Discounting Associated With Health Behaviors: Ecological Momentary Assessment Study

**DOI:** 10.2196/48954

**Published:** 2024-02-27

**Authors:** Amanda Luken, Jill A Rabinowitz, Jonathan L Wells, David W Sosnowski, Justin C Strickland, Johannes Thrul, Gregory D Kirk, Brion S Maher

**Affiliations:** 1 Department of Mental Health Bloomberg School of Public Health Johns Hopkins University Baltimore, MD United States; 2 Department of Epidemiology School of Population Health Virginia Commonwealth University Richmond, VA United States; 3 Department of Psychiatry and Behavioral Sciences School of Medicine Johns Hopkins University Baltimore, MD United States; 4 Sidney Kimmel Comprehensive Cancer Center Johns Hopkins University Baltimore, MD United States; 5 Centre for Alcohol Policy Research La Trobe University Melbourne Australia; 6 Department of Epidemiology Bloomberg School of Public Health Johns Hopkins University Baltimore, MD United States; 7 School of Medicine Johns Hopkins University Baltimore, MD United States

**Keywords:** delay discounting, measurement, Monetary Choice Questionnaire, ecological momentary assessment, substance use, substance abuse, questionnaire, validity, validation, measurement, monetary, reward, rewards, survey, mobile phone

## Abstract

**Background:**

Delay discounting quantifies an individual’s preference for smaller, short-term rewards over larger, long-term rewards and represents a transdiagnostic factor associated with numerous adverse health outcomes. Rather than a fixed trait, delay discounting may vary over time and place, influenced by individual and contextual factors. Continuous, real-time measurement could inform adaptive interventions for various health conditions.

**Objective:**

The goals of this paper are 2-fold. First, we present and validate a novel, short, ecological momentary assessment (EMA)–based delay discounting scale we developed. Second, we assess this tool’s ability to reproduce known associations between delay discounting and health behaviors (ie, substance use and craving) using a convenience-based sample.

**Methods:**

Participants (N=97) were adults (age range 18-71 years), recruited on social media. In phase 1, data were collected on participant sociodemographic characteristics, and delay discounting was evaluated via the traditional Monetary Choice Questionnaire (MCQ) and our novel method (ie, 7-item time-selection and 7-item monetary-selection scales). During phase 2 (approximately 6 months later), participants completed the MCQ, our novel delay discounting measures, and health outcomes questions. The correlations between our method and the traditional MCQ within and across phases were examined. For scale reduction, a random number of items were iteratively selected, and the correlation between the full and random scales was assessed. We then examined the association between our time- and monetary-selection scales assessed during phase 2 and the percentage of assessments that participants endorsed using or craving alcohol, tobacco, or cannabis.

**Results:**

In total, 6 of the 7 individual time-selection items were highly correlated with the full scale (*r*>0.89). Both time-selection (*r*=0.71; *P*<.001) and monetary-selection (*r*=0.66; *P*<.001) delay discounting rates had high test-retest reliability across phases 1 and 2. Phase 1 MCQ delay discounting function highly correlated with phase 1 (*r*=0.76; *P*<.001) and phase 2 (*r*=0.45; *P*<.001) time-selection delay discounting scales. One or more randomly chosen time-selection items were highly correlated with the full scale (*r*>0.94). Greater delay discounting measured via the time-selection measure (adjusted mean difference=5.89, 95% CI 1.99-9.79), but not the monetary-selection scale (adjusted mean difference=–0.62, 95% CI –3.57 to 2.32), was associated with more past-hour tobacco use endorsement in follow-up surveys.

**Conclusions:**

This study evaluated a novel EMA-based scale’s ability to validly and reliably assess delay discounting. By measuring delay discounting with fewer items and in situ via EMA in natural environments, researchers may be better able to identify individuals at risk for poor health outcomes.

## Introduction

### Background

Delay discounting quantifies a subject’s relative preference for more smaller, immediate rewards over large, delayed rewards. Stated another way, delay discounting can be defined as the perceived value of a reward based on the temporal delay in the receipt of the reward. Greater delay discounting (ie, preference for smaller, short-term rewards over larger, long-term rewards) [1,2] increases the risk for several health conditions, including obesity [3], gambling disorder [4], anxiety, depression [5], substance use disorder [6], and poor substance use disorder treatment response [7-10]. Although some research describes delay discounting as an immutable trait [11], findings from laboratory and clinical studies suggest delay discounting varies by place, time, and other contextual factors [12,13]. Delay discounting’s malleability may represent a promising avenue for interventions or treatment to reduce the risk for myriad negative health outcomes. In this study, we present a novel tool to rapidly assess delay discounting in remote studies and natural environments. Below, we describe traditional methods to measure delay discounting, the potential for ecological momentary assessment (EMA) to improve delay discounting measurement, and this study’s goals.

### Traditional Measurement of Delay Discounting

Delay discounting rates quantify consequence devaluation (eg, a monetary reward’s devaluation) by delay [14]. One method to calculate delay discounting rates considers the devaluation process as a hyperbolic decay [15], as shown in equation 1.

*V* = *A* / (1 + *kD*) **(1)**

*V* represents the indifference point or a small immediate reward’s value, *A* represents the long-term reward value, *k* represents the delay discounting rate, and *D* represents the long-term reward’s delay (ie, long-term reward’s waiting time). Short-term reward preference corresponds to a greater delay discounting rate (ie, future rewards’ steeper devaluation by delay), whereas long-term reward preference corresponds to a shallower delay discounting rate.

Researchers often measure delay discounting with the Monetary Choice Questionnaire (MCQ) [16-19]. To obtain the delay discounting rate, the MCQ asks participants to choose between hypothetical smaller, sooner monetary rewards and various larger, later rewards varied by magnitude and delay [16,18]. Researchers often use the 27-item MCQ due to its intuitive administration and straightforward calculations [16]. There are numerous MCQ variations, including a 21-item version [17] and a 9-item version [19].

Although many studies use the MCQ, a continuous indifference point measure may better capture delay discounting rates due to reduced ceiling effects and faster data saturation. The MCQ has been criticized as constraining delay discounting rates, resulting in ceiling effects in populations with high delay discounting rates [18]. For example, although the MCQ was originally tested with people who use heroin, the MCQ’s current versions limit comparisons across or within populations because people who use drugs can reach maximum delay discounting rates (ie, ceiling effects) [18]. Directly measuring indifference points along a continuous measure (eg, how long are you willing to wait to receive US $100 instead of US $30 today?) may prevent ceiling effects. Some studies have implemented a continuous measure for the indifference point [20], but few have used EMA-based continuous measures. A continuous indifference point measure increases the set of possible discounting rate estimates [20] and may capture the delay discounting rate with fewer items than the MCQ. Repeatedly administering the MCQ may fatigue participants [21], but a continuous indifference point measure may allow researchers to quickly reach data saturation (eg, obtaining consistent delay discounting rates over fewer repeated measures) [20].

### EMA of Delay Discounting

EMA-based delay discounting research can measure behavior in context, abbreviate delay discounting scales, and minimize bias compared to more traditional data collection methods. First, EMA methods collect repeated measures on participants’ behaviors in situ [22] throughout the day, capturing ephemeral behaviors and moods traditional methods may miss or mismeasure [23]. Indeed, EMA may identify place and time cues that increase the risk for greater delay discounting (ie, preference for sooner, smaller rewards) and increased maladaptive behavior risk [24]. For example, one EMA study identified a relationship between delay discounting and acute substance use withdrawal [25]. The study found constant MCQ scores for the first few hours after substance use, but MCQ scores peaked 4-6 hours after alcohol and cannabis use and after 2 hours for stimulant use [25]. Second, repeated delay discounting assessments may shorten scales with good reliability. Fewer questions varied in presentation may reduce study participation burden and fatigue [21] and straightlining (ie, giving the same answer to all questions) [26]. Other researchers have shortened existing delay discounting assessments to reduce participant burden [18], but EMA-based scales could further abbreviate scales with good reliability. Finally, EMA may have less bias than traditional data collection. Less latency between the event occurrence and survey completion relative to traditional methods may reduce recall bias and measurement error [27]. Capturing delay discounting in situ may also decrease social desirability bias associated with stigmatized behaviors (eg, substance use) compared to face-to-face interviews [27].

The goals of this paper are 2-fold. First, we present and validate a novel, short, EMA-based delay discounting scale we developed. Second, we assess this tool’s ability to reproduce known associations between delay discounting and health behaviors (ie, substance use and craving) using a convenience-based sample.

## Methods

### Participants

We posted study advertisements on Facebook and Instagram via a Facebook profile. Phase 1 study advertisements appeared on mobile and desktop newsfeeds from December 2020 to February 2021. Study advertisements directed participants to “MetricWire” (MetricWire Inc), a digital research platform. Participants were instructed to download MetricWire’s iPhone- and Android-compatible smartphone app. We administered all surveys via MetricWire, a widely used research app designed for in-the-moment, contextual data collection. Study staff verified participants’ email addresses and phone numbers. Then, interested participants created a password-protected account and answered the screener in the app. Throughout the study, participants could anonymously contact study staff with questions or concerns through the app’s instant messaging (IM) system. Researchers emailed and invited phase 1 participants to join phase 2 in July 2021.

US residents aged 18 years or older with smartphones were eligible to participate. Eligible participants read study and consent materials through the MetricWire app. To ensure participants understood the study objectives, participants had 3 attempts to correctly answer 3 multiple-choice questions regarding the study’s purpose, length, and potential risks. Study incentives and anonymous recruitment risked individuals feigning their country of residence and reregistering under fake accounts. As a result, MetricWire removed participants with IP addresses already registered or abroad.

### Ethical Considerations

This study received institutional review board approval from Johns Hopkins Bloomberg School of Public Health (IRB00011160). All participants provided informed consent and were informed they would receive US $10 for the completion of phase 1 and a maximum of US $75 for the completion of phase 2. The data analyzed were anonymous and deidentified.

### Sample Size

For a diverse sample, we sought to equally recruit participants from 6 categories based on age (ie, 18-30, 31-50, and >50 years) and race (eg, White and non-White). In recruitment, we considered adults identifying as 2 or more races as non-White adults. We as aimed to recruit 33 adults for each quota, or 198 participants in total. These 198 participants would yield approximately 2821 observations (198 participants × [1 baseline survey + (6 days × 3 follow-up surveys)], assuming 75% compliance).

### Study Design

We used a 2-phase EMA study via MetricWire’s smartphone app. In phase 1, participants completed the 8-item MCQ, a sociodemographic questionnaire, and our 2 novel 7-item continuous delay discounting surveys. Researchers invited participants who completed phase 1 to participate in phase 2 a few months later. Phase 2 comprised a survey on the first day (baseline) and 3 daily follow-up surveys on their smartphone for 6 consecutive days, amounting to 19 total surveys. Participants received the 3 follow-up surveys on their smartphones at random times between their self-reported wake and sleep times. In phase 2, the baseline survey included the original MCQ, health behavior assessments (eg, substance use and craving), and our 2 novel EMA-based delay discounting measures. At each daily follow-up, participants completed our EMA-based delay discounting tool and a health outcomes questionnaire (eg, past-hour substance use and craving). The phase 2 baseline survey duration approximated 20 minutes, and each follow-up survey approximated 5 minutes.

Participants received compensation based upon adherence. Phase 1 completion renumerated participants with US $10 credit for Tango, a third-party gift card provider. In phase 2, participants received US $20 Tango credit for baseline assessment completion, US $5 credit each day if they completed at least 1 follow-up survey, and US $25 bonus credit for completing at least 75% (14/19) of surveys upon the study’s end. Participants received links to their accrued credit through the app’s IM system. To improve adherence, text in the consent form encouraged participants to enable MetricWire notifications on their smartphones and to request technical support through the app’s IM system. Halfway through phase 2, study staff messaged participants their adherence rate and a reminder about the US $25 bonus credit.

### Measures

#### Time- and Monetary-Selection Measures

We presented participants with 2 continuous delay discounting measures. For the first type, participants chose how long they would wait to receive the long-term reward rather than the short-term reward (eg, *D* from equation 1), hereafter referred to as *time-selection items*. In the second type, participants chose how much money they would need to receive today rather than the long-term reward (eg, *V* from equation 1), hereafter referred to as *monetary-selection items*. Textbox 1 outlines the time-selection and monetary-selection items.

**Modified ecological momentary assessment (EMA)–based time-selection and monetary-selection delay discounting items for a 2-phase EMA study assessing the validity of a novel delay discounting measure. Items were randomized during the study.**

**Time-selection items (participants could choose between 0 and 52 weeks)**

- How long would you be willing to wait to get US $100 instead of US $50 today?
- How long would you be willing to wait to get US $100 instead of US $70 today?
- How long would you be willing to wait to get US $100 instead of US $10 today?
- How long would you be willing to wait to get US $100 instead of US $80 today?
- How long would you be willing to wait to get US $100 instead of US $40 today?
- How long would you be willing to wait to get US $100 instead of US $30 today?
- How long would you be willing to wait to get US $100 instead of US $99 today?

**Monetary-selection items (participants could choose between US $1 and US $99)**

- How much money would you take today instead of US $100 in a year?
- How much money would you take today instead of US $100 in 1 month?
- How much money today would you take today instead of US $100 in 6 months?
- How much money would you take today instead of US $100 in 3 months?
- How much money would you take today instead of US $100 in 2 weeks?
- How much money would you take today instead of US $100 in 1 week?
- How much money would you take today instead of US $100 tomorrow?

**Figure 1:**
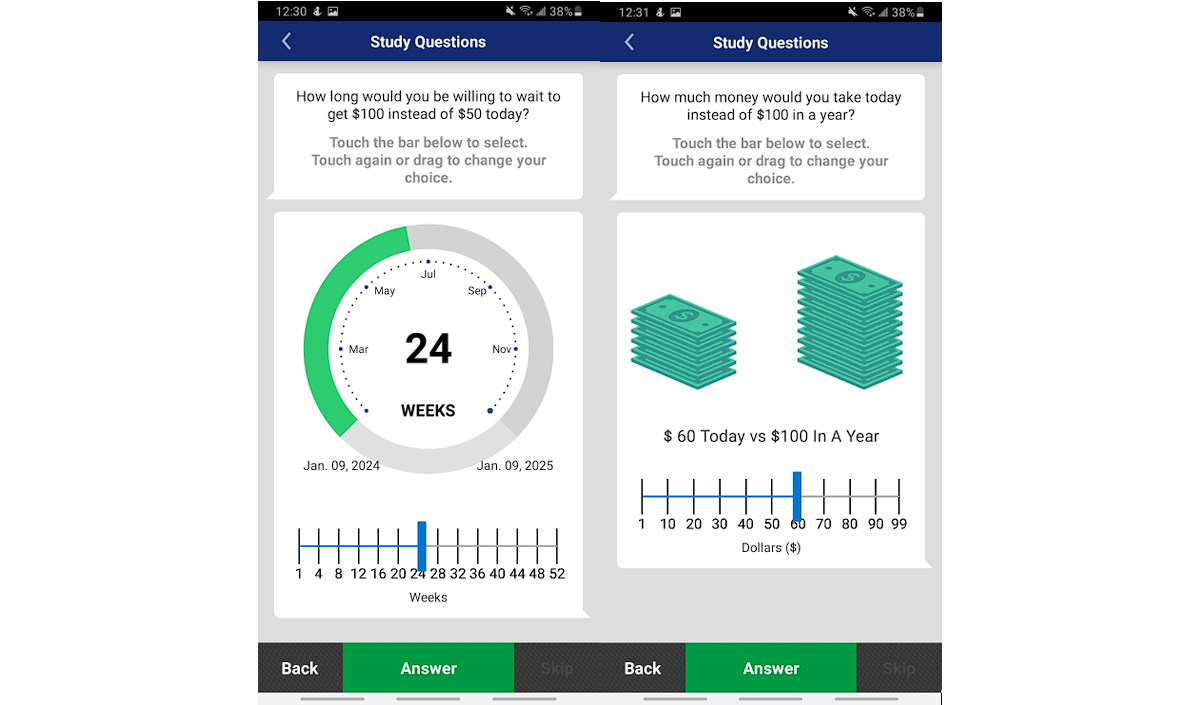
Time-selection item (left) and a monetary-selection item (right) examples for a 2-phase ecological momentary assessment study assessing the validity of a novel delay discounting measure.

Figure 1 displays the time- and monetary-selection items’ in-app appearance. Participants used a sliding scale to select their wait time or monetary reward. Our novel measures comprised 7 time-selection and 7 monetary-selection items. Participants answered time- and monetary-selection items at phase 1, phase 2 baseline, and phase 2 follow-up. From the measured indifference point and long-term reward delay, the delay discounting rate (*k*) was directly calculated via equation 1, since we knew *V* (the immediate reward’s value), *A* (the delayed reward’s value), and *D* (the time delay). The geometric mean across all items was then calculated to determine the average delay discounting rate for the time- and monetary-selection items.

#### Original MCQ

The original MCQ was administered in both phase 1 and 2 baseline surveys to validate our time- and monetary-selection measures. Participants answered 7 “Would you rather receive US $50 today or US $100 in 1 year?” variants. The long-term reward consistently displayed US $100, but the time delay and short-term reward amount varied. The time delay ranged from 2 months to a year, while the short-term reward amount ranged from US $10 to US $99. See Multimedia Appendix 1 for all administered items.

#### Substance Use

To determine our novel measures’ predictive use and ability to reproduce established associations between delay discounting and a health condition, we asked participants if they used or craved substances within the last hour during phase 2. Surveyed substances included (1) alcohol; (2) tobacco, cigarettes, cigarillos, cigars, vaping, and nicotine; (3) cannabis, marijuana, pot, grass, and hash; (4) cocaine, coke, and crack; (5) prescription stimulants (eg, Ritalin and Concerta); (6) methamphetamines (eg, speed, crystal meth, and ice); (7) inhalants (eg, nitrous oxide and glue); (8) sedatives of sleeping pills (eg, Valium); (9) hallucinogens (eg, lysergic acid diethylamide and acid); (10) street opioids (eg, heroin); (11) prescription opioids (eg, fentanyl); and (12) others. We created individual-level variables reflecting the total percentage of phase 2 surveys endorsing using or craving alcohol, cannabis, or tobacco. We did not examine other use or craving of any other substance with very low base rates.

### Statistical Analysis

#### Overview

Prior to conducting analyses, we examined the time- and monetary-selection items’ distribution. Participants identified as outliers or who provided invalid responses (ie, always selecting maximum or minimum values) were dropped. Additionally, we examined the delay discounting distributions for violations of normality and the need for log transformations.

The study objectives included identifying noninformative items in our time- and monetary-selection scales, examining scale stability across phase 1 and 2 baselines, validating time- and monetary-selection items with the phase 1 and 2 baseline MCQ, and determining the minimally sufficient set of time- and monetary-selection items required to capture delay discounting. All analyses were performed in R (version 4.0.4; R Foundation for Statistical Computing).

#### Objective 1: Identify Noninformative Items Within New Measures

We examined Pearson correlations between the phase 1 geometric average delay discounting rate of the 7-item time-selection scale and each time-selection item’s delay discounting rate. This was repeated for the monetary-selection items in phase 1. Then, we repeated the item-scale correlations separately for time- and monetary-selection items in phase 2. Items uncorrelated with the full scale were then dropped from analyses.

#### Objective 2: Examine New Measures’ Test-Retest Reliability

For the second objective, we compared the delay discounting function from phase 1 baseline to phase 2 baseline via Pearson correlations.

#### Objective 3: Validate New Measures

For the third goal, we compared time- and monetary-selection scales to the study’s gold standard—the traditional MCQ—via Pearson correlations. The correlation between the delay discounting rate derived from phase 1’s traditional MCQ and the delay discounting rate derived from both the time- and monetary-selection tools at phase 1 and phase 2 was examined. The correlation between the delay discounting rate derived from phase 2’s traditional MCQ and the time- and monetary-selection tools at phase 2 was additionally assessed.

#### Objective 4: Shorten New Scale

For the fourth goal, we iteratively examined the Pearson correlation between the geometric average delay discounting rate of a randomly chosen set of the informative items and the full 7-item scale using phase 1 data.

#### Objective 5: Assess the Association Between New Delay Discounting Measures and Substance Use

Finally, we tested the association between delay discounting and substance use to assess our time- and monetary-selection measures’ predictive use. Linear regression analyses were conducted to examine associations between the predictors (ie, phase 2 baseline delay discounting rates calculated from our time- and monetary-selection measures) with 6 outcomes (ie, percentage of assessments participants reported using or craving alcohol, cannabis, or tobacco). Participant’s age, sex, and completed number of surveys were adjusted. We mean-centered continuous predictor variables and conducted analyses with completed surveys only.

## Results

### Overview

**Figure 2:**
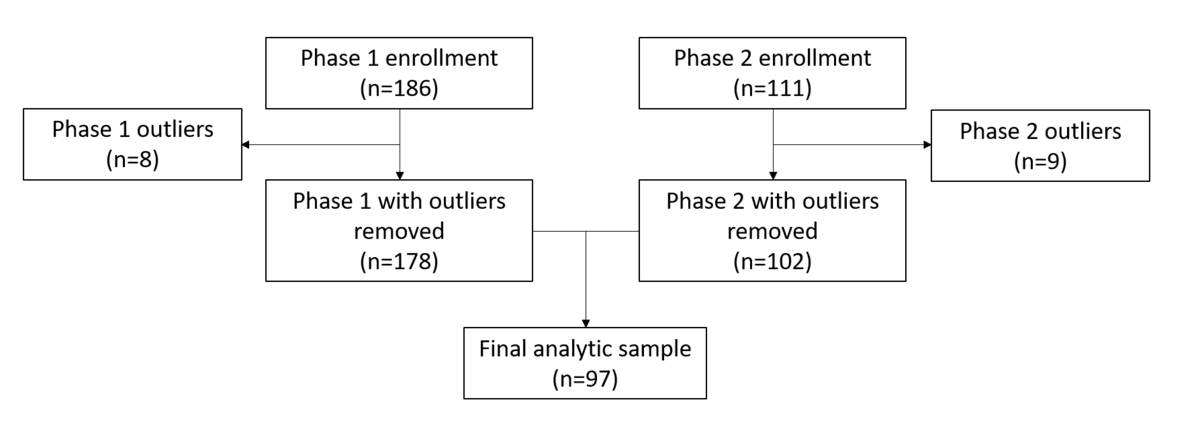
Analytical sample flowchart for a 2-phase ecological momentary assessment (EMA) study assessing the validity of a novel delay discounting measure. Participants were recruited from Facebook and Instagram to participate in a mobile-based EMA study.

In phase 1, a total of 186 participants were recruited, of whom 111 agreed to participate in phase 2. To identify potential outliers, the delay discounting rate’s SD was calculated at each phase separately for the 7 time- and monetary-selection responses. The 4 resulting plots were then visually inspected. From the phase 1 monetary-selection geometric average delay discounting rate distribution, 6 data points were identified as outliers that were 3 SDs from the standardized mean. For phase 1 time-selection delay discounting items, individuals who consistently selected only the minimum time delay (1 day, n=2) or the maximum time delay (52 days, n=1) were removed. We excluded 8 total participants, noting them as outliers, from our phase 1 analytical sample. For phase 2 time-selection delay discounting items, 3 individuals were identified as outliers that were 3 SDs above the standardized mean—1 individual consistently selected the minimum time delay (1 day) and 2 individuals consistently selected the maximum time delay (52 days). For phase 2 monetary-selection delay discounting items, 5 individuals were excluded as outliers that were 3 SDs above the standardized mean. The phase 2 raw sample included 9 outliers in total. The 8 participants from phase 1 and 9 participants from phase 2 were excluded from the analyses. Our analytic sample included individuals with valid phase 1 and phase 2 data, resulting in a sample of 97 participants (Figure 2).

There were some differences in demographic characteristics between those who completed phase 1 only versus those in our analytic sample who completed phases 1 and 2. In particular, there was a greater proportion of individuals who identified as African American or Black who completed phase 1 only (33/89, 37%) compared to those who completed phases 1 and 2 (9/97, 9%; ^2^_2_=21.4; *P*<.001). A greater proportion of individuals identified as White (55/97, 57%), Asian (25/97, 26%), or 2 or more races (33/97, 34%) who completed phases 1 and 2 relative to phase 1 only (White: 39/89, 44%; Asian: 12/86, 14%; 2 or more races: 8/97, 8%). There were no differences in terms of the completion of the study based on participant sex, ethnicity, or income.

Among participants who completed phase 1 and phase 2 (ie, our analytic sample; N=97), most participants identified as female (n=72, 74%) and as White (n=56, 58%). Approximately 80% (n=79) of participants were 50 years and younger of age, and 70% (n=68) reported an annual income below US $75,000 (Table 1). On average, participants completed 16 (SD 4) of 19 surveys. On average, 4% (SD 9%; n=0.8) of a given participant’s surveys endorsed alcohol use, 6% (SD 21%; n=1.22) endorsed tobacco use, 7% (SD 22%; n=0.004) endorsed craving tobacco, and 8% (SD 18%; n=1.60) endorsed craving alcohol.

**Table 1:** Characteristics of US adults participating in a novel 2-phase ecological momentary assessment–based study assessing the validity of a novel delay discounting tool, 2020-2021 (N=97).

Characteristics				Values
**Sex, n (%)**				
	Male			25 (26)
	Female			72 (74)
**Race, n (%)**				
	Asian			24 (25)
	Black			9 (9)
	White			56 (58)
	2 or more races			8 (8)
**Age group (years), n (%)**				
	18-25			28 (29)
	26-35			25 (26)
	36-50			26 (27)
	51-70			16 (17)
	>71			2 (2)
**Income (US $), n (%)**				
	<$30,000			30 (31)
	$30,000-$49,999			19 (20)
	$50,000-$74,999			19 (20)
	$75,000-$100,000			19 (20)
	>$100,000			10 (10)
**Number of surveys completed per person**				
	Mean (SD)			16 (4)
	Range			1-19
**Percentage of surveys reflecting substance use or craving**				
	**Alcohol use**			
		Mean (SD)	4 (9)	
		Range	0-53	
	**Cannabis use**			
		Mean (SD)	4 (14)	
		Range	0-73	
	**Tobacco use**			
		Mean (SD)	6 (21)	
		Range	0-100	
	**Alcohol craving**			
		Mean (SD)	8 (18)	
		Range	0-100	
	**Cannabis craving**			
		Mean (SD)	8 (20)	
		Range	0-100	
	**Tobacco craving**			
		Mean (SD)	7 (22)	
		Range	0-100	

The delay discounting rate distribution generated from the time- and monetary-selection items and the original MCQ, which manifested a positively skewed distribution, was inspected. Thus, the delay discounting rates were log-transformed to obtain normally distributed variables for analyses examining the relationship between delay discounting and other measured characteristics. Below, we present findings from the study’s 5 objectives.

### Objective 1: Identify Noninformative Items Within New Measures

The Pearson correlations between each item’s delay discounting rate and the 7-item scale’s geometric average delay discounting rate were examined. In phase 1, we found high correlations (*r* range=0.89-0.96) for 6 of the 7 time-selection items (Multimedia Appendix 2). The poorly performing item (*r*=0.38 in phase 1) asked participants to choose between US $99 today or US $100 in the future. The small difference in the immediate versus delayed reward may have yielded poor discrimination between individuals with high versus low discounting. In subsequent analyses, this item was dropped. We reproduced the finding in phase 2 (Multimedia Appendix 3)—6 of the items strongly correlated with the scale (*r* range=0.88-0.94), and phase 1’s poor-performing item continued to perform poorly in phase 2 (*r*=0.35 in phase 2). In phase 1, a relatively low correlation was found (*r* range=0.45-0.85) between individual money-selection items and the 7-item scale’s geometric average delay discounting rate (Multimedia Appendix 4). Moreover, the item-to-scale correlations were not consistent between phase 1 and phase 2, and only 3 items were consistently correlated (*r*>0.75) across phases 1 and 2 (Multimedia Appendix 5).

### Objective 2: Examine New Measures’ Test-Retest Reliability

Delay discounting rates from phase 1 to phase 2 for both the novel time- and monetary-selection measures were compared. We found moderate to high correlations between phase 1 and 2 time-selection (*r*=0.66; *P*<.001) and money-selection delay discounting (*r*=0.41; *P*<.001), indicating good and acceptable test-retest reliability, respectively (Table 2).

**Table 2:** Correlations of log-transformed delay discounting variables across 2 phases of a mobile-based, ecological momentary assessment study assessing the validity of a novel delay discounting tool between 2020 and 2021 (N=97).

Variables		Phase 1 MCQ^a^	Phase 1 time-selection delay discounting variable	Phase 1 monetary-selection delay discounting variable	Phase 2 MCQ	Phase 2 time-selection delay discounting variable	Phase 2 monetary-selection delay discounting variable
**Phase 1 MCQ**							
	*r*	1	0.76	0.57	0.51	0.45	0.22
	*P* value	—^b^	<.001	<.001	<.001	<.001	.03
**Phase 1 time-selection delay discounting variable**							
	*r*	0.76	1	0.66	0.65	0.71	0.38
	*P* value	<.001	—	<.001	<.001	<.001	<.001
**Phase 1 monetary-selection delay discounting variable**							
	*r*	0.57	0.66	1	0.42	0.49	0.42
	*P* value	<.001	<.001	—	<.001	<.001	<.001
**Phase 2 MCQ**							
	*r*	0.51	0.65	0.42	1	0.72	0.50
	*P* value	<.001	<.001	<.001	—	<.001	<.001
**Phase 2 time-selection delay discounting variable**							
	*r*	0.45	0.71	0.49	0.72	1	0.41
	*P* value	<.001	<.001	<.001	<.001	—	<.001
**Phase 2 monetary-selection delay discounting variable**							
	*r*	0.22	0.38	0.42	0.50	0.41	1
	*P* value	.03	<.001	<.001	<.001	<.001	—

^a^MCQ: Monetary Choice Questionnaire.

^b^Not available.

### Objective 3: Validate New Measures

**Figure 3:**
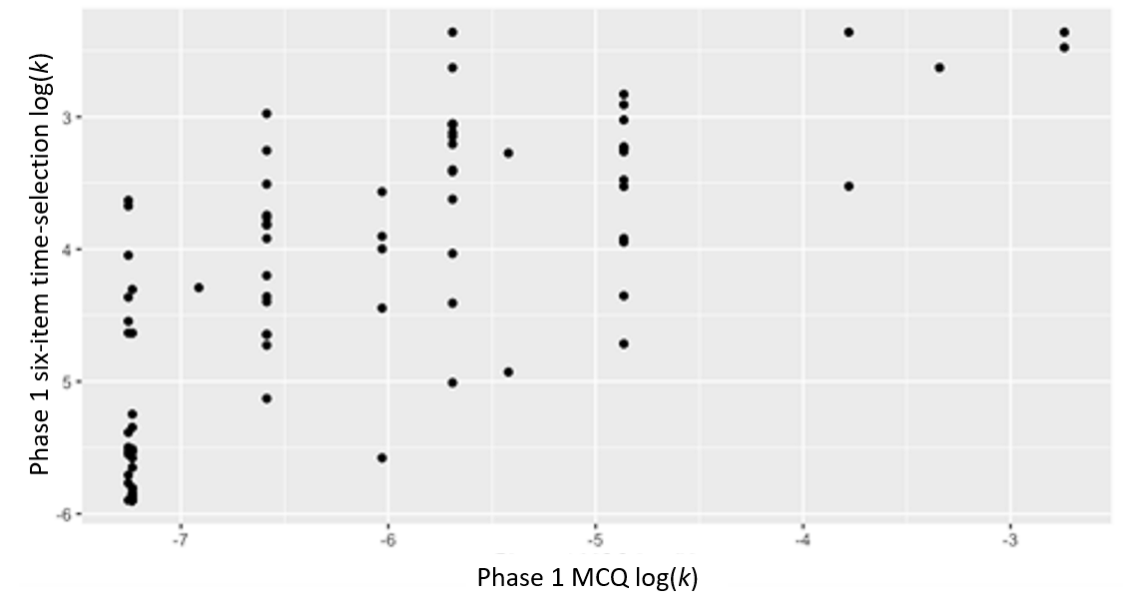
Correlations between phase 1 MCQ log(delay discounting rate) and phase 1 six-item time-selection log(delay discounting rate). Data are from a mobile-based ecological momentary assessment study assessing the validity of a novel delay discounting tool across 2 phases between 2020 and 2021. Participants were US adults recruited from social media. An example of a time-selection item is “How long would you be willing to wait to get US $100 instead of US $50 today?” MCQ: Monetary Choice Questionnaire.

**Figure 4:**
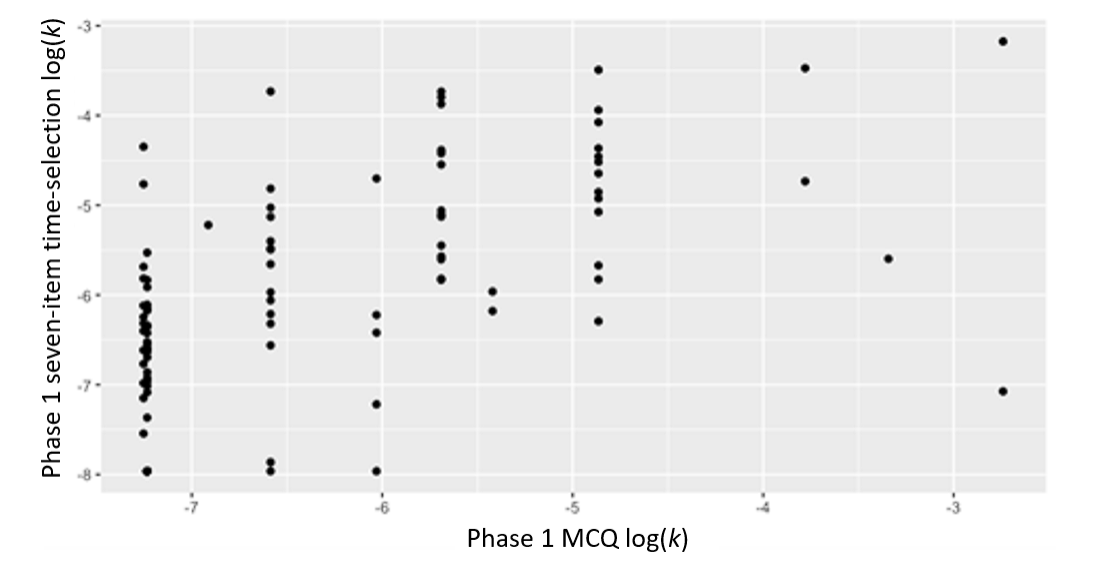
Correlations between phase 1 MCQ log(delay discounting rate) and phase 1 seven-item monetary-selection log(delay discounting rate). Data are from a mobile-based ecological momentary assessment study assessing the validity of a novel delay discounting tool across 2 phases between 2020 and 2021. Participants were US adults recruited from social media. An example of a monetary-selection item is “How much money would you take today instead of US $100 in a year?” MCQ: Monetary Choice Questionnaire.

The correlations between the traditional MCQ and the novel time- and monetary-selection approaches were examined (Table 2). Excellent and acceptable correlations were observed between the phase 1 traditional MCQ–derived delay discounting rate with the phase 1 time-selection delay discounting rate (*r*=0.76; *P*<.001; Table 2 and Figure 3) and the phase 2 time-selection delay discounting rate (*r*=0.45; *P*<.001), respectively. Moderate to small positive correlations were noted between the phase 1 MCQ–derived delay discounting rate with phase 1 monetary-selection delay discounting rate (*r*=0.57; *P*<.001; Figure 4) and phase 2 money-selection delay discounting rate (*r*=0.22; *P*=.03). The phase 2 traditional MCQ delay discounting rate was highly positively associated with the phase 2 time-selection (*r*=0.72; *P*<.001) and moderately positively associated with the monetary-selection delay discounting rate (*r*=0.50; *P*<.001).

### Objective 4: Shorten New Scale

**Figure 5:**
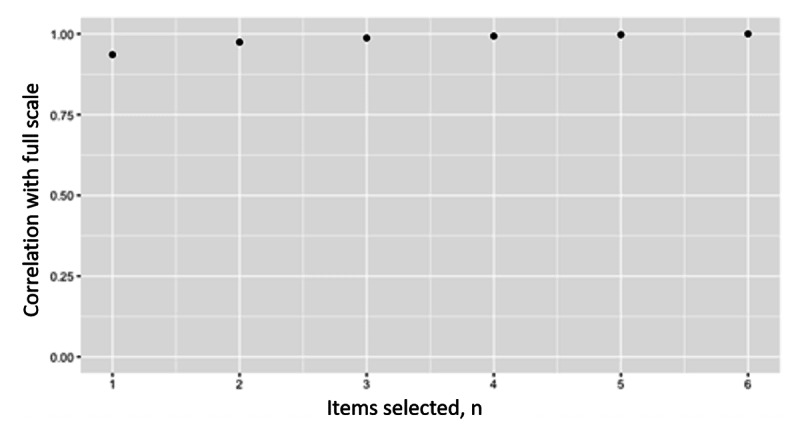
Correlations between phase 1 six-item time-selection log(delay discounting rate) and randomly selected subscales of 1 to 6 items. Data are from a mobile-based ecological momentary assessment study assessing the validity of a novel delay discounting tool across 2 phases between 2020 and 2021. Participants were US adults recruited from social media. An example of a time-selection item is “How long would you be willing to wait to get US $100 instead of US $50 today?”.

**Figure 6:**
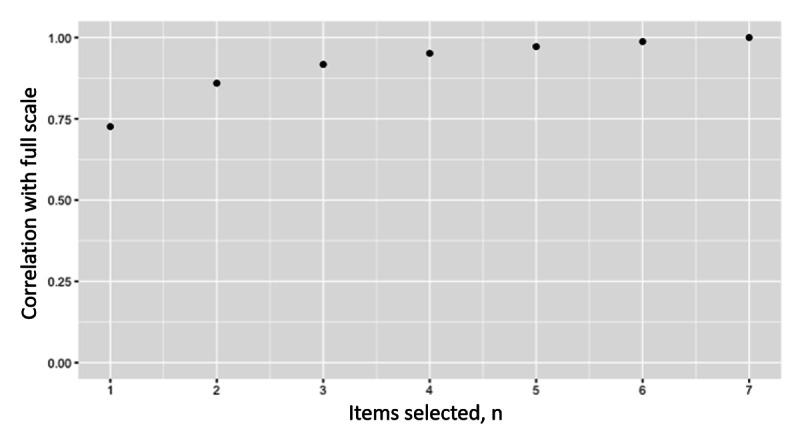
Correlations between phase 1 seven-item monetary-selection log(delay discounting rate) and randomly selected subscales of 1 to 7 items. Data are from a mobile-based ecological momentary assessment study assessing the validity of a novel delay discounting tool across 2 phases between 2020 and 2021. Participants were US adults recruited from social media. An example of a monetary-selection item is “How much money would you take today instead of US $100 in a year?”.

We also examined if an abbreviated scale could approximate the full 6-item scale of the time-selection approach. To construct the abbreviated scale, we randomly selected N (between 1 and 5) items across 100 iterations at each N and calculated the correlation between the N item’s geometric average delay discounting rate and the full 7-item time-selection scale’s geometric average delay discounting rate. A high correlation was found between randomly selected 1 (*r*=0.94) or 2 (*r*=0.97) item scales and the full 6-item scale, indicating a relatively small number of items can approximate the full scale (Figure 5). The analyses were repeated with the monetary-selection approach, and a relatively weak correlation was found between randomly selected 1 (*r*=0.74) or 2 (*r*=0.86) item scales and the full 7-item scale (Figure 6).

### Objective 5: Assess the Association Between New Delay Discounting Measures and Substance Use

Participants with greater phase 2 baseline delay discounting rates from the MCQ and our time-selection measure had a higher proportion of surveys endorsing tobacco craving and use. There were trend-level significant associations between the original MCQ delay discounting rate (adjusted mean difference=3.46, 95% CI –0.63 to 7.55; *P*=.10) and the time-selection delay discounting rate with tobacco craving (adjusted mean difference=3.92, 95% CI –0.12 to 7.95; *P*=.06; Table 3). Significant associations were observed between the original MCQ delay discounting rate (adjusted mean difference=4.32, 95% CI 0.29-8.34; *P*=.04) and time-selection delay discounting rate (adjusted mean difference=5.89, 95% CI 1.99-9.79; *P*=.003) with the percentage of surveys endorsing tobacco use. No significant associations were observed between the phase 2 baseline monetary-selection delay discounting rate and substance use or craving (all *P*>.05). There were no significant differences between alcohol or cannabis use or craving and the delay discounting rate derived from both the MCQ and our novel measures (all *P*>.05).

**Table 3:** Associations among phase 2 log-transformed delay discounting measures with the percentage of surveys endorsing alcohol, cannabis, or tobacco use or craving (N=97).^a^

Measures and variable		Adjusted mean difference (95% CI)^b^	*P* value
**Phase 2 baseline MCQ^c^**			
	Percentage of alcohol use	1.00 (–0.75 to 2.75)	.26
	Percentage of cannabis use	0.20 (–2.80 to 3.20)	.89
	Percentage of tobacco use	4.32 (0.29 to 8.34)	.04
	Percentage of alcohol craving	2.73 (–0.84 to 6.31)	.13
	Percentage of cannabis craving	1.02 (–3.16 to 5.20)	.63
	Percentage of tobacco craving	3.46 (–0.63 to 7.55)	.10
**Phase 2 baseline time-selection delay discounting**			
	Percentage of alcohol use	0.64 (–1.10 to 2.38)	.47
	Percentage of cannabis use	–1.07 (–4.04 to 1.89)	.47
	Percentage of tobacco use	5.89 (1.99 to 9.79)	.003
	Percentage of alcohol craving	2.65 (–0.89 to 6.20)	.14
	Percentage of cannabis craving	–0.36 (–4.51 to 3.78)	.86
	Percentage of tobacco craving	3.92 (–0.12 to 7.95)	.06
**Phase 2 baseline monetary-selection delay discounting**			
	Percentage of alcohol use	0.97 (–0.27 to 2.22)	.13
	Percentage of cannabis use	–1.17 (–3.30 to 0.97)	.28
	Percentage of tobacco use	–0.62 (–3.57 to 2.32)	.68
	Percentage of alcohol craving	1.78 (–0.79 to 4.34)	.17
	Percentage of cannabis craving	–1.29 (–4.27 to 1.69)	.39
	Percentage of tobacco craving	–1.07 (–4.03 to 1.90)	.48

^a^Data are from a mobile-based ecological momentary assessment study assessing the validity of a novel delay discounting tool between 2020 and 2021. Participants were US adults recruited from social media. An example of a time-selection item is “How long would you be willing to wait to get US $100 instead of US $50 today?” An example of a monetary-selection item is “How much money would you take today instead of US $100 in a year?”

^b^Linear regression models adjusted for number of completed surveys and participant sex and age. Delay discounting rates are log-transformed.

^c^MCQ: Monetary Choice Questionnaire.

## Discussion

### Principal Findings

In this study, a novel EMA-based tool was developed to overcome the limitations of existing delay discounting measures. In contrast to traditional delay discounting measures captured at a single time point (eg, the MCQ), our EMA-based delay discounting measure used a continuous indifference point to minimize ceiling effects, abbreviate delay discounting scales, capture state-like fluctuations in delay discounting, and reduce measurement error. We showed that a time-selection, EMA-based method could accurately assess delay discounting. In addition, the novel tool successfully reproduced some associations between delay discounting and substance use behaviors observed in the literature, specifically the established association between delay discounting and tobacco use.

To develop the EMA-based scale, we analyzed the scales’ reliability across 2 phases and its validity in comparison to the MCQ. In total, 6 of the 7 time-selection items correlated with the full scale. We dropped the uninformative, 7th time-selection item—“How long would you be willing to wait to get US $100 instead of US $99 today?” The US $1 difference may have failed to provide sufficient response variability, because even short delays (eg, 1 week) would result in devaluing US $100 over US $99 [28]. Overall, the time-selection EMA measure reliably and validly measured delay discounting.

However, the monetary-selection items did not consistently contribute to delay discounting measurement. There are a number of reasons to explain this lack of performance of this measure. First, we believe participants simply may have struggled to decide which amount they would take today over the longer-term reward. Second, the monetary-selection measure comprised fuzzy units (eg, cost of one’s patience for a specific period), and studies have shown fuzzy units increase short-term reward preferences compared to discrete units (eg, how long one would wait) [29,30]. Participants may not have as much behavioral experience to quantify the abstract monetary cost to wait for an already specified duration. Participants, however, may reliably draw from personal experience and past behaviors to assess their capacity for patience, as conveyed in the time-selection items. Third, framing time as a date (as in the time-selection items) or as days (as in the monetary-selection items) may have influenced participants’ delay discounting rates [30]. Finally, monetary-selection items may have needed a larger, long-term reward to capture variability in long-term reward devaluation. When asked to wait a year to receive US $100, participants choose a short-term reward as close as possible to the long-term reward, but if given a larger, long-term reward, participants may choose a smaller, short-term reward relative to the long-term reward [31].

After assessing the measures’ validity and reliability, we determined that randomly selecting 1 to 2 time-selection items could sufficiently capture delay discounting. Similarly, another study also captured delay discounting with 2 items with no sensitivity loss [32]. An abbreviated EMA-based scale can minimize participant fatigue and sustain participant attention during data collection.

Additionally, our tool successfully reproduced some established associations between delay discounting and substance use or craving. Based on the time-selection items and the original MCQ, tobacco use and cravings often increased with greater delay discounting. Other studies have similarly evidenced a preference for smaller, immediate rewards over larger, long-term rewards (ie, greater delay discounting) when involving nicotine use [33,34]. On the other hand, we found no relationship between delay discounting and alcohol or cannabis use or craving. Other studies have found positive, modest associations between delay discounting and alcohol and marijuana use and craving in other samples [35-37]. Similarly, a recent meta-analysis showed a small association between delay discounting and cannabis use [38].

### Limitations

Selection and response biases limit the generalizability of our study’s findings for a number of reasons. First, our study sample differs from our target population with regard to age, race, and sex. We created age and race recruitment quotas to avoid immigrative selection bias, but we ultimately did not meet the quota for older adults identifying as non-White, and we unintentionally oversampled female participants. Other studies similarly had greater ease recruiting White older adults over non-White older adults on social media [39,40]. This study, however, successfully recruited participants across income levels, which may improve generalizability to more socioeconomically diverse populations. Our sample also exceeded other measurement development studies’ sample sizes [41]. Second, few participants endorsed substance use. Our study advertisements did not target individuals who use substances, which likely contributed to low substance use endorsement in our sample and limited ability to identify associations between delay discounting and alcohol and cannabis use and craving. Our advertisement strategy, however, demonstrated our measures’ use for the general population and minimized participant self-selection by substance use frequency. Moreover, we did not incentivize substance use reporting, which may have failed to counter social desirability bias and underreporting, but our strategy improved our confidence in minimal false reports [42]. Finally, our analyses examining the association between delay discounting and substance use and craving did not account for temporality, limiting our ability to draw causal inferences regarding the directionality of effects. Future studies should examine the tool’s use in more specific health populations, such as individuals who use substances to excess or engage in other addictive behaviors, and examine the causal relationship between the new tool and substance use.

### Conclusions

We found that 1 or 2 randomly selected items from our novel EMA-based time-selection measure can sufficiently assess delay discounting. Our abbreviated EMA-based scale may overcome data collection barriers related to participants’ attentional capacity and measurement barriers due to ceiling effects [18]. Beyond aiding delay discounting–specific research, our transdiagnostic tool may help with intervention assessment and rapid detection of individuals at risk for specific health conditions. In terms of intervention assessment, delay discounting may serve as an outcome for measuring the effectiveness and efficacy of behavior change interventions. For example, an intervention targeting binge eating may seek to assess the intervention’s momentary effect on how an individual values the immediate reward of binge eating over the long-term health benefits of abstaining from binge eating. In this case, delay discounting may serve as a rapid proxy to assess the intervention’s effect on reward valuation. Additionally, our tool may also help detect individuals at higher risk in a high-risk population associated with greater impulsivity. For example, one study suggested delay discounting tools may be adapted to discern HIV risk among individuals with and without cocaine use disorder [43]. Future studies should test the time-selection measures’ predictive use in clinical, high-risk populations.

## Appendix

Multimedia Appendix 1 Monetary Choice Questionnaire–administered items.

Multimedia Appendix 2 Phase 1 time-selection per-item log(delay discounting rate) correlation with full 7-item log(delay discounting rate).

Multimedia Appendix 3 Phase 2 time-selection per-item log(delay discounting rate) correlation with full 7-item log(delay discounting rate).

Multimedia Appendix 4 Phase 1 monetary-selection per-item log(delay discounting rate) correlation with full 7-item log(delay discounting rate).

Multimedia Appendix 5 Phase 2 monetary-selection per-item log(delay discounting rate) correlation with full 7-item log(delay discounting rate).

## Abbreviations

EMA: ecological momentary assessment
IM: instant messaging
MCQ: Monetary Choice Questionnaire

## Data Availability

The data sets generated and analyzed during this study are available from the corresponding author on reasonable request.
