# Supplementary material for: Designing and Validating a Novel Method for Assessing Delay Discounting Associated With Health Behaviors: Ecological Momentary Assessment Study
Source: JMIR Form Res. 2024 Feb 27;8:e48954. doi: 10.2196/48954 (PMC10933719; doi:10.2196/48954)
Supplement: Multimedia Appendix 1 [file formative_v8i1e48954_app1.docx]

| Items |
| --- |
| 1. Would you rather receive $50 today or $100 in a year? |
| 1. Would you rather receive $70 today or $100 in 1 month? |
| 1. Would you rather receive $100 in 6 months of $10 today? |
| 1. Would you rather receive $80 today or $100 in 6 months? |
| 1. Would you rather receive $100 in 1 month or $40 today? |
| 1. Would you rather receive $100 in 2 weeks or $30 today? |
| 1. Would you rather receive $100 in 2 weeks of $99 today? |
| 1. Would you rather receive $100 in 1 month or $80 today? |
