# Supplementary figures and images for: Designing and Validating a Novel Method for Assessing Delay Discounting Associated With Health Behaviors: Ecological Momentary Assessment Study
Source: JMIR Form Res. 2024 Feb 27;8:e48954. doi: 10.2196/48954 (PMC10933719; doi:10.2196/48954)

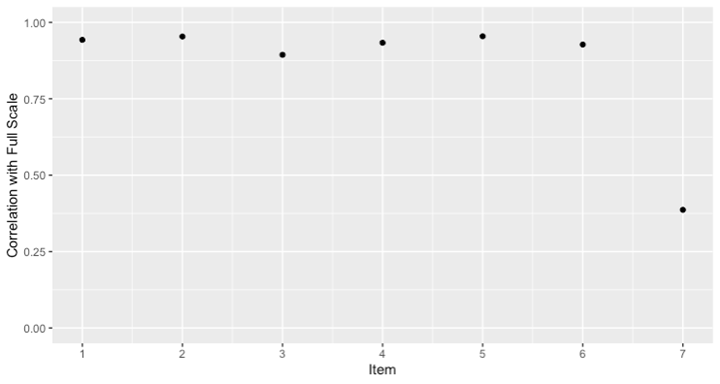

Supplement: Multimedia Appendix 2 [file formative_v8i1e48954_app2.png]

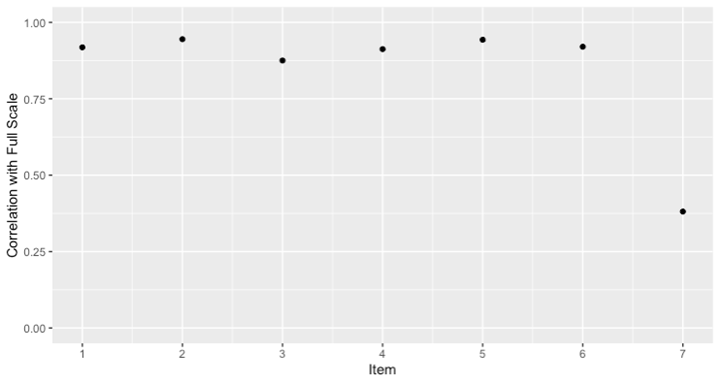

Supplement: Multimedia Appendix 3 [file formative_v8i1e48954_app3.png]

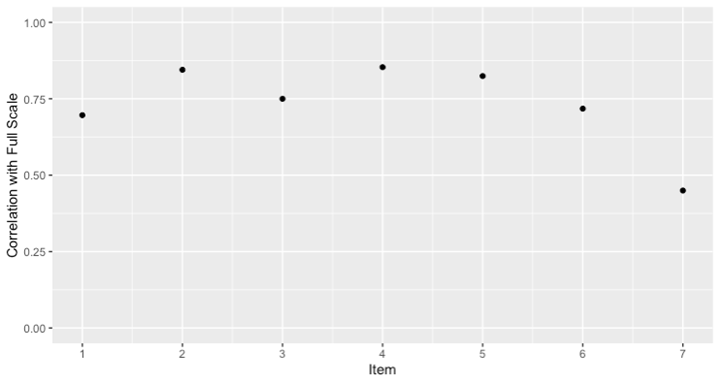

Supplement: Multimedia Appendix 4 [file formative_v8i1e48954_app4.png]

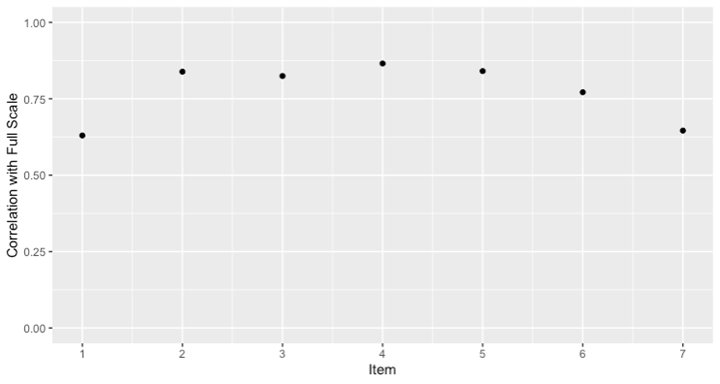

Supplement: Multimedia Appendix 5 [file formative_v8i1e48954_app5.png]
